# Mistakes are not an option: aggression from peers and other correlates of anxiety and depression in pediatricians in training

**DOI:** 10.3389/fpsyg.2024.1346530

**Published:** 2024-07-18

**Authors:** María Yoldi-Negrete, Diana Guízar-Sánchez, Rebeca Robles-García, Carlos-Alfonso Tovilla-Zárate, Ricardo-Arturo Saracco-Álvarez, Iñaki Navarro-Castellanos, Ana-Carolina Hill-de-Titto, Ana Fresán

**Affiliations:** ^1^Subdirección de Investigaciones Clínicas, Instituto Nacional de Psiquiatría Ramón de la Fuente Muñíz, Mexico City, Mexico; ^2^Departamento de Fisiología de la Facultad de Medicina, Universidad Nacional Autónoma de México, Mexico City, Mexico; ^3^Centro de Investigación en Salud Mental Global, Instituto Nacional de Psiquiatría Ramón de la Fuente Muñíz, Mexico City, Mexico; ^4^División Académica Multidisciplinaria de Comalcalco, Universidad Juárez Autónoma de Tabasco, Tabasco, Mexico; ^5^Departamento de Cardiología Pediátrica, Hospital Regional Lic, Adolfo López Mateos, Instituto de Seguridad y Servicios Sociales de los Trabajadores del Estado, Mexico City, Mexico; ^6^Departamento de Educación de Pre y Posgrado, Hospital Infantil de México Federico Gómez, Mexico City, Mexico

**Keywords:** pediatricians in training, depression, anxiety, perfectionism, patient death

## Abstract

**Introduction:**

Pediatricians in training are a population at risk for anxiety and depression: they face long working hours, they are confronted with the suffering and death of a vulnerable population and may have personal characteristics that put them in danger. Nonetheless, few studies have looked at their prevalence and associated factors. We aimed to compare demographic, professional activities, adversities and perfectionism personality features between a group of pediatricians in training with depression and/or anxiety and a group of pediatricians in training without depression nor anxiety and, to identify the variables that increase the probabilities of depression and/or anxiety in pediatricians in training.

**Methods:**

Pediatric residents who voluntarily answered an online survey distributed via personal and institutional social media channels from October 2019 to April 2021, as part of a cross-sectional study with medical specialists and residents from Mexico were included. Demographic information, professional activities and adversities, perfectionism personality features (Multidimensional Perfectionism Scale), depression and anxiety (ICD-11 PHC) were evaluated. For comparison purposes Chi-square tests (χ^2^) and independent sample *t*-tests were used. A logistic regression analysis was used to identify those variables that increase the probabilities of depression and/or anxiety.

**Results:**

934 pediatric residents answered the survey. 11.6% reported having depression and 20% anxiety. Being single, a history of anxious or depressive episodes, being the resident in charge of a patient who died, aggressions from colleagues and a high concern for errors were associated with current depression and/or anxiety.

**Discussion:**

Screening for depressive and anxious symptoms should be routinely performed from entry to the residency to favor early intervention. A shift from promoting perfectionism to a more compassionate training could serve a population facing so many adversities.

## 1 Introduction

According to the World Health Organization (WHO), a mental disorder is characterized by a clinically significant disturbance in an individual’s cognition, emotional regulation or behavior. Anxiety and depressive disorders being the most common mental disorders (World Health Organization, 2023). The WHO states that during a depressive episode, the person experiences feelings of sadness, irritability, emptiness and loss of pleasure or interest in activities, for most of the day, almost every day, for at least two weeks. Anxiety disorders are characterized by excessive fear and worry, as well as behavioral disturbances, distress or major impairment of functioning. Depression and anxiety are more prevalent among physicians; the lifetime risk of depression among the general population is of 9% for men and 15% for women, while for physicians it is suggested to be 15% for men and 20–30% for women (Tyssen et al., 2000; Alonso et al., 2004). Suicide is a leading cause of mortality among physicians and is the only cause of death for which the risk is twice as high than among the general population (Ryan et al., 2023).

Depression and anxiety are common in the field of medical specialties, especially during residency, with prevalence estimates of depression ranging from 20.9 to 43.2% depending on the instrument used for assessment (Mata et al., 2015; de Sá et al., 2023). Dyrbye et al. (2014) in a national study of U.S. residents from various specialties found that 50.8% screened positive for depression. In a 2014 systematic review by Hope and Henderson (2014) in the United States, the prevalence of anxiety was found to be 7.7 to 65.5% and of depression, 6 to 66.5%. Sepúlveda-Vildosola et al. (2012) reported a prevalence of 39.6% for depression and 29.6% for anxiety in pediatric residents, with a coexistence of both pathologies in 21.4%. In 2022, Martínez-García et al. (2022) reported 17% of anxiety and 45% of depression in medical specialties, particularly anesthesiology, internal medicine and pediatrics, the latter also with the highest rates of anxiety.

The presence of these conditions is not without concern as they take a significant toll on the quality of life of physicians on the short and long-term, as depressive episodes can be recurrent and are related to increased disability (World Health Organization, 2023). The presence of depression and anxiety can go as far as reducing their life expectancy by increasing the risk of suicide (Jiménez-López et al., 2015; Braquehais et al., 2016; Gold and Schwenk, 2020). Furthermore, the mental well-being of physicians can have notorious impact on patient outcomes: in a scoping review with a qualitative method, Sattar et al. (2023) found that poor mental wellness (identified as burnout, depression, disappointment, depersonalization and conscientiousness) had an inverse association with medical professionalism (empathy, academic performance, compassion and unprofessional behavior). In addition, in a prospective cohort study of pediatric residents, Brunsberg et al. (2019) examined the relationship between depression, burnout and medical errors, with an emphasis on harmful versus non harmful errors: they found that depressed physicians had a 3-fold higher rate of harmful errors compared to those who screened negative for depression.

Physicians in training are a population with unique characteristics that might explain the increased prevalence of depression and anxiety, especially since longitudinal studies show a significant increase in depressive symptoms after the start of the residency. Medical residents deal with challenges unique to their line of work, including coping with patient’s suffering, life-threatening circumstances, and death, in addition to tight work schedules (Mata et al., 2015). In Mexico, depending on the institution, the specialty of pediatrics is obtained after a 3 to 4-year residency program, following medical school. Students in this residency program, like those in others, alternate between attending lectures and practicing medicine under the supervision of senior residents and attending physicians, in a highly demanding schedule. Until 2022, in Mexico there was no regulation on working hours for residents, and the current legislation states only broad guidelines for days on-call without specifications of total hours worked per-week or in a given day (Alcocer Varela, 2022) and the fact that there is a small doctor/patient ratio (49.55/100,000 inhabitants) in our country implies heavy work loads.

Besides working conditions, the learning environment is deeply attached to residents´ wellbeing. The power hierarchies and teaching through humiliation in medical residencies, can lead to a shame response in learners, who then constantly compare one’s behavior against standards one has come to believe and make learning from errors difficult (Edmondson, 2004; Robertson and Long, 2019). The culture of perfectionism in medicine, characterized by a low tolerance for mistakes and a high sense of commitment to the patients, is generally promoted and highly reinforced in general culture: as an example, Rizo et al. (2002) gathered responses from physicians, nurses and patients to the question “What’s a good doctor and how do you make one?”; several connotations to perfectionism can be found in these answers. Evidently, the internalization of these cultural expectations is high: socially-prescribed perfectionism is one of the most important predictors of psychological distress in medical students (Henning et al., 1998; Thomas and Bigatti, 2020). Moreover, pediatricians might be at an even higher risk of perfectionism, as they specialize in dealing with life-threatening circumstances in children which we, as humans, are naturally prone to protect (Martin et al., 2023). How much perfectionism serves its purpose – improving the ability to care for these children – remains to be determined. Meanwhile, maladaptive perfectionism which can be reflected in high levels of self-criticism, suffering from negative evaluation, and concerning about mistakes, has a positive correlation with depression, and could represent an underlying factor for the increased prevalence of depression in this population (Wei et al., 2021).

Given the importance of pediatricians in our society as being the primary caregivers of our children and adolescents’ health, we aimed: (1) to compare demographics, professional activities, self-reported adversities and perfectionism personality features between a group of pediatricians in training with depression and/or anxiety and a group of pediatricians in training without depression nor anxiety, and (2) to identify the variables that increase the probabilities of depression and/or anxiety in pediatricians in training. We decided to perform the present study with a group of pediatricians in training with depression and/or anxiety and those without any of these conditions, as affected individuals with one condition frequently present significant symptoms of the other, making the comorbidity between the two among the most common in psychiatry. Also, many symptoms overlap.

## 2 Materials and methods

### 2.1 Participants

The present study was part of a cross-sectional study conducted on a sample of medical specialists and specialty residents from Mexico who completed an online survey (Fresán et al., 2023). Participants were initially recruited through a convenience sampling approach by being contacted by the researchers and subsequently asked to disseminate the online survey within their medical networks. Also, an invitation from the Ramón de la Fuente Muñiz National Institute of Psychiatry (INPRFM) was circulated by email and social media. Finally, the Division of Postgraduate Studies of the National Autonomous University of Mexico (UNAM) helped by contacting possible candidates. The invitations from both institutions explained the aims and procedures of the study. The approval of the study by the Ethics and Research Committees, stated the anonymity of the surveys’ responses (no identifying information was requested), and the personal decision to withdraw from the study by dropping out of the survey. Recruitment was undertaken from October 2019 to April 2021.

Due to its exploratory nature and to the best of our knowledge, being the first study aimed at evaluating variables related to the mental health of medical specialists and residents in the country, this convenience sampling method was considered the most appropriate; it sampled those medical specialists and residents whose medical practice or training was currently located in Mexico (homogeneous feature) and who were available and willing to participate. The key advantages of the convenience sampling are that it is unexpensive, efficient and simple to implement (Jager et al., 2017). For this study, we focused only on the sample of pediatric residents who responded the online survey.

### 2.2 Assessment procedures

To capture online survey responses we used RSForm!Pro in a Joomla! based website (both under GNU General Public License), which was accessible only through a link that directed participants to the content of the survey, and was not available for general visitors. The website is run by a non-profit organization focused on the dissemination of information regarding psychiatry. The complete survey comprises around 15 pages, however, for the present study only the variables described below were analyzed. JAVAScripts for mandatory items were used for completeness checks and were signaled to the users throughout the survey. Given that all items were marked as mandatory, only completely answered surveys could be submitted and therefore no survey with missing data was analyzed. Submission was not possible unless all mandatory items had been answered. A combination of birthdate and IP address was used to identify duplicates and when found, only the most recent entry was retained. Cookies were not used. Information regarding the aims and procedures of the study, and the guarantee of anonymity were included on the first page of the survey. The survey was conducted in Spanish and the following four sections of the complete online survey were used:

1. Demographic information: Included variables such as age at the time of the study, sex, and marital status.
2. Professional activities and self-reported adversities: This section included the maximum hours per day spent on activities related to their profession (the continuous on-call shift was not included), and the year of residency they were currently enrolled on.

An assessment of self-reported adversities since the beginning of the residency was performed. Participants were asked if they had been aggressed (physically, verbally, or psychologically), the role of the assailant (patient, patient’s relative, colleague/senior resident), and if they had been the physician in charge of a patient who died (not by suicide). These questions intended to evaluate their professional life as residents.

1. Perfectionism personality features: The Multidimensional Perfectionism Scale (MPS-F) validated in Mexican population (Paredes et al., 2010), was used to assess the perfectionism personality features of the participants. The scale comprises 35 self-reported items answered on a Likert agreement scale (1 = totally disagree to 5 = totally agree) that evaluates five main dimensions of perfectionism: Concern for errors (CE), Organization (O), Indecision of action (IA), Personal standards (PS) and Expectations (E). Items in the Expectations dimensions were rewritten to assess the expectations of others about being a medical specialist (Guízar-Sánchez et al., 2020). A total score is obtained with the sum of the dimensions’ scores. Higher scores (dimensions and total score) reflect higher perfectionism.
2. Mental health: The ICD-11 PHC screening test was used, which includes five items for the Anxiety Scale and five items for the Depression Scale. A score > 3 on each scale has a positive predictive value of 84% for the cases with a current clinical diagnosis of depression and 89% of the cases with a clinical diagnosis of anxiety in the Mexican population (Goldberg et al., 2017). These cut-off points were used to divide our sample into those pediatric residents with and without depression and/or anxiety.

Residents were asked if they had had previous anxious and/or depressive episodes, which were answered in a yes/no format.

Current perceived work-related distress was assessed on a 100-point visual analog scale, where “0” reflected not experiencing any perceived work-related distress and “100” the maximum possible perceived work-related distress. The specific question for evaluating perceived work-related distress was “What level of distress do you think you have as a result of your profession?.” Visual analog scales have been widely used in behavioral and social sciences to measure subjective states including work-related distress (Dutheil et al., 2022).

### 2.3 Ethical considerations

The study was approved by the Ethics and Research Committees of the INPRFM (CONBIOETICA-09-CEI-010_20170316) and UNAM (FM/DI/075). Duplicate entries were identified through participants’ date of birth and IP address and eliminated from the dataset. To maintain anonymity, the dates of birth and IPs of the remaining participants were also eliminated. As noted above, participants took part in this study on a voluntary basis, were not paid for their participation, and could withdraw their consent by not completing the online survey.

### 2.4 Statistical analyses

Descriptive information was obtained by frequencies and percentages for categorical variables while means and standard deviations (S.D.) were determined for continuous variables. Variables included in the four assessed sections were compared between pediatric residents with depression and/or anxiety, using the previously described cutoff points, and those without these mental health problems using Chi-square tests (χ2) and independent sample *t*-tests were used where appropriate to fulfill the first objective of the study.

Demographic features (age at the time of the study, sex, and marital status), professional activities (maximum hours per day spent on activities related to their profession), self-reported adversities (being attacked, the identity of the assailant and if they had been the attending physician of a patient who died – not by suicide), perfectionism personality features (concern for errors, organization, indecision of action, personal standards and expectations), previous anxious or depressive episodes and current perceived work-related distress were included as possible variables that increase the probabilities of depression and/or anxiety in a multivariate logistic regression analysis using the backward stepwise modeling approach (second objective study). To determine the goodness of fit of the model, the Hosmer and Lemeshow test was used while the Nagelkerke R^2^ value was used to determine the proportion of variation in the dependent variable (current depression and/or anxiety) explained by the variables included in the models. Two models are reported: the first one, containing all the previously mentioned variables, and a final model including only the variables that remained significant after the backward stepwise process. This final model was selected in accordance with the most adequate goodness of fit index displayed and the highest variance explained. Alpha value for tests was set at *p* < 0.05. The SPSS version 21 for Windows PC was used to perform the analyses.

## 3 Results

### 3.1 Demographic features

A total of 934 pediatric residents answered the online survey. According to the register of the Division of Postgraduate Studies of UNAM, our sample represents around 78.2% of the registered pediatric residents (*n* = 1,196 during the study assessment). A higher percentage of the participants were women (69.4%, *n* = 648) and single (56.7%, *n* = 530) and the mean age of the sample was 28.9 years (S.D. = 2.1, range 25–39).

From the total sample, 24.1% (*n* = 225) reported having depression and/or anxiety according to the ICD-11 PHC screening test, 11.6% (*n* = 108) with current depression, 20% (*n* = 187) anxiety and 7.5% (*n* = 70) met both criteria for depression and anxiety. No differences between pediatric residents with and without depression and/or anxiety were found in terms of sex or age. Nevertheless, a higher percentage of pediatric residents with depression and/or anxiety were single (i.e., not in a relationship akin to marriage) (see Table 1).

**Table 1:** Demographics, professional activities, and adversities between pediatric residents with and without depression and/or anxiety.

	Total *n* = 934	Without depression nor anxiety *n* = 709	With depression and/or anxiety *n* = 225	Statistics
*Demographic features n %*				
Sex – Women	648 69.4	487 68.7	161 71.6	χ^2^ = 0.6, *p* = 0.41
Age – years (mean; S.D.)	28.9 2.1	28.9 2.1	28.9 2.2	*t* = −0.08, *p* = 0.92
Marital status – Single^+^	404 43.3	388 54.7	142 63.1	χ^2^ = 4.8, *p* = 0.02
*Professional activities and self-reported adversities n %*				
Year of residency				χ2 = 1.4, *p* = 0.68
1st	77 8.2	56 7.9	21 9.3	
2nd	398 42.6	306 43.2	92 40.9	
3rd	412 44.1	314 44.3	98 43.6	
4th	47 5.0	33 4.7	14 6.2	
Maximum working hours per day (mean; S.D.)	12.8 5.4	12.3 6.2	13.2 4.5	*t* = −2.1, *p* = 0.03
Aggressions – Yes	377 40.4	252 35.5	125 55.6	χ^2^ = 28.4, *p* < 0.001
Physical – Yes	366 39.2	250 35.3	116 51.6	χ^2^ = 19.0, *p* < 0.001
Verbal – Yes	337 36.1	226 31.9	111 49.3	χ^2^ = 22.5, *p* < 0.001
Psychological – Yes	369 39.5	249 35.1	120 53.3	χ^2^ = 23.7, *p* < 0.001
Committed by patients – Yes	244 26.1	160 22.6	84 37.3	χ^2^ = 19.2, *p* < 0.001
Committed by patient’s relative – Yes	266 28.5	174 24.5	92 40.9	χ^2^ = 22.4, *p* < 0.001
Committed by colleague/resident higher rank – Yes	172 18.4	98 13.8	74 32.9	χ^2^ = 41.3, *p* < 0.001
Resident in charge of a patient who died – Yes	468 50.1	335 47.2	133 59.1	χ^2^ = 9.6, *p* = 0.002

*Single includes single, divorced and separated participants, in contrast to married participants and participants living together in a relationship akin to marriage.

### 3.2 Professional activities and self-reported adversities

Most of the pediatric residents were in the second (42.6%, *n* = 398) or third year (44.1%, *n* = 412) of their residency, 8.2% (*n* = 77) were in their first year and the remaining 5.0% (*n* = 47) in their fourth year of residency. Excluding the 36-h continuous on-call shift, the mean maximum hours per day spent on professional activities was 19.6 (S.D. = 4.7, range 8–24). Half of the residents reported they had been the resident in charge of a patient who died – not by suicide. Less than half of the residents reported being aggressed during their residency (40.4%, *n* = 377). Psychological and physical aggression were the most common forms of attack, particularly from patients’ relatives and patients.

When professional activities and self-reported adversities were compared between residents with and without depression and/or anxiety, no differences emerged between residents regarding the year of residency.

Almost 60% of residents with depression and/or anxiety reported being the resident in charge of a patient who died, a higher percentage when compared to those residents without depression nor anxiety (47.2%).

In general, being aggressed was also more frequent in those residents with depression and/or anxiety (55.6% vs. 35.5%), with higher percentages of verbal, physical and psychological violence (almost half or just over half of residents with depression and/or anxiety vs. less than 40% in those without depression nor anxiety). Consistent with this result, patients’ relatives, followed by patients and colleagues, were the main perpetrators of the attacks with higher percentages reported when compared to those residents without depression nor anxiety.

Professional activities and adversities of the total sample and between groups are displayed in Table 1.

### 3.3 Perfectionism personality features and mental health

Pediatric residents with depression and/or anxiety display higher perfectionism with a higher total score on the MPS-F scale. The dimensions where perfectionism was higher in these participants were: concern for errors, indecision of action, and expectations (see Table 2). As seen in Table 2, nearly twice as many residents with current depression and/or anxiety report having had previous episodes of depression and/or anxiety (66.7% vs. 34%) and display higher levels of perceived work-related distress.

**Table 2:** Perfectionism personality features and self-perceived distress.

	Total *n* = 934	Without depression nor anxiety *n* = 709	With depression and/or anxiety *n* = 225	Statistics
Perfectionism dimensions and self-perceived distress Mean S.D.				
MPS-F perfectionism scale				
Concern for errors	36.0 10.3	35.3 10.6	38.1 9.0	*t* = −3.5, *p* < 0.001
Organization	23.3 3.9	23.4 3.7	23.0 4.6	*t* = 1.4, *p* = 0.14
Indecision of action	16.9 4.6	16.7 4.9	17.6 3.8	*t* = −2.6, *p* = 0.008
Personal standards	15.3 2.8	15.3 2.7	15.5 3.0	*t* = −0.8, *p* = 0.38
Expectations	13.1 3.2	13.0 3.2	13.5 3.0	*t* = −2.3, *p* = 0.02
Total	104.8 19.4	103.9 19.7	107.9 18.2	*t* = −2.7, *p* = 0.007
Mental health, *n* %				
Previous episodes of depression and/or anxiety – Yes	391 41.9	241 34.0	150 66.7	χ^2^ = 74.9, *p* < 0.001
Perceived work-related distress (mean; S.D.)	54.1 28.2	50.5 28.3	65.4 24.9	*t* = −7.0, *p* < 0.001

### 3.4 Factors associated with depression and/or anxiety in pediatric residents

The proportion of variation explained by the two multivariate regression models was of 24.3%. In the first and the final multivariate regression models (after the backward stepwise process), the most important variables associated with the presence of depression and/or anxiety in pediatric residents were previous episodes of depression and/or anxiety and having been aggressed by a colleague/senior resident. These variables display odds ratios (OR) higher than three and two, respectively. Being single and being the resident in charge of a patient who died were also associated to current depression and/or anxiety with an OR of 1.49 and 1.52, respectively. A higher concern for errors (perfectionism) and a higher level of perceived work-related distress were also related to the presence of depression and/or anxiety. Although their OR were just over the value of one, their contribution to the final model was important (concern for errors R^2^ = 4%; work-related distress R^2^ = 3.9%). On the other hand, a higher organization (perfectionism) was the only variable associated with a decreased probability of pertaining to the depression and/or anxiety group, but its contribution to the variance of the final model was low (R^2^ = 0.8%). Table 3 shows the results of the initial and final regression models.

**Table 3:** Factors associated with depression and/or anxiety in pediatric residents.

	Initial Model			Final Model		
	OR	95% C.I.		OR	95% C.I.	
Sex – Women	1.08	0.74	1.56	-	-	-
Age	0.96	0.85	1.08	-	-	-
Marital status – single	1.53*	1.06	2.19	1.49*	1.05	2.10
Year of residency – 2nd and 3rd year	0.99	0.68	1.45	-	-	-
Maximum working hours per day	0.97	0.94	1.01	0.97	0.94	1.01
Physical attacks – Yes	0.52	0.09	2.88	0.53	0.26	1.05
Verbal attacks– Yes	1.29	0.55	2.99	-	-	-
Psychological attacks – Yes	0.81	0.12	5.28	-	-	-
Aggression committed by patients – Yes	1.50	0.88	2.55	1.48	0.88	2.50
Aggression committed by patient’s relative – Yes	1.53	0.86	2.71	1.61	0.95	2.74
Aggression committed by colleague/resident of higher rank – Yes	2.38***	1.44	3.93	2.39***	1.46	3.89
Being the resident in charge of a patient who died – Yes	1.57**	1.10	2.22	1.52**	1.07	2.14
Perfectionism – Concern for errors	1.04**	1.01	1.08	1.05***	1.03	1.07
Perfectionism – Organization	0.94	0.89	1.002	0.94*	0.90	0.98
Perfectionism – Indecision of action	1.02	0.97	1.07	-	-	-
Perfectionism – Personal standards	0.96	0.87	1.05	-	-	-
Perfectionism – Expectations	1.03	0.95	1.11	-	-	-
Previous episodes of depression and/or anxiety – Yes	3.08***	2.18	4.36	3.04***	2.16	4.29
Perceived work-related distress	1.01***	1.01	1.02	1.01***	1.01	1.02
Constant	0.06			0.03		
Initial Model: R^2^ Nagelkerke = 0.24 Test Hosmer and Lemeshow = *p* = 0.92 Final Model: R^2^ Nagelkerke = 0.24 Test Hosmer and Lemeshow = *p* = 0.94						

*p ≤ 0.05, **p ≤ 0.01***p ≤ 0.001. Hyphen (−) indicates variables which did not enter in the regression model.

## 4 Discussion

The aim of the present study was to compare demographic, professional activities, adversities and perfectionism personality features between a group of pediatricians in training with depression and/or anxiety and a group of pediatricians in training without depression nor anxiety and, to identify the variables that increase the probabilities of depression and/or anxiety in pediatricians in training. Our findings show that a significant percentage of pediatric residents in our sample could be classified as having depression and/or anxiety. From the various factors which would likely be associated with depression and/or anxiety, being single, having a history of anxious and/or depressive episodes, perfectionism, work-related distress, being the attending physician of a patient who died and aggression from colleagues were the most relevant.

The percentage of pediatricians in training with depression and/or anxiety (11.6 and 20% respectively) was significantly higher than the reported prevalence of depression and anxiety in the general population in Mexico (2.96 and 3.85% respectively) (Institute for Health Metrics and Evaluation [Internet], 2023). There are very few studies assessing depression or anxiety in the field of pediatrics, as most have focused on burnout or were conducted exclusively during the COVID-19 pandemic. However, Brunsberg et al. (2019) conducted a prospective study from 2011 to 2013 where 537 pediatrics residents from the US and Canada were screened for depression and burnout. Twenty percent of their sample screened positive for depression. Moreover, screening positive for depression was associated with a three-fold higher rate of harmful errors, a finding that is important to keep in mind as depression in physicians is relevant from multiple perspectives. Nevertheless, the relationship between these variables is highly complex: as Lawson (2020) points out in response to the previous study, the interpretation of the association between mistakes and depression should be cautios and rather than increasing stigma and deepening the gap between clinical onset and treatment, these findings should help to recognize that caring for the mental health of physicians is in society’s best interest. In this regard, our findings further demonstrate the importance of depression and anxiety prior to medical residency, as will be next addressed.

Personal vulnerability is a major factor in the development of mental health problems. The numerous studies that have identified past mental conditions as a risk factor for current depressive episodes, anxiety or other mental health problems have led to new policies for early identification of people at risk in the medical environment and to encourage physicians to seek help (Center et al., 2003; Eckleberry-Hunt and Lick, 2015). Detecting those who have had depressive episodes or significant anxiety before entering a medical residency seems indeed one of the most important measures we can take to ensure early intervention. Screening for depression and anxiety is not a difficult task; the true barrier is stigma, which remains high among physicians and the medical culture, and is not far from another prominent feature of this culture: perfectionism (Aaronson et al., 2018).

Perfectionism as a personality trait might precede medical school, but is likely to be nurtured by medical culture (Eley et al., 2022). Pediatric residents build an identity that values perfection after immersing and socializing in the pediatric culture (Thomas and Bigatti, 2020). Perfectionism can trigger shame when one fails to meet an ideal standard or is criticized or judged by others (Robertson and Long, 2019), a common scenario in medicine where teaching by shaming is highly prevalent (Fnais et al., 2014; Roberts, 2023). This perfectionist trait, as predicted by Robertson and Long (2019) is an important factor explaining the presence of depressive and anxious symptoms in our sample. This is consistent with the findings from other studies linking perfectionism to burnout in pediatricians (Martin et al., 2022), and to other negative mental health outcomes in other health care providers (O’Brien and Page, 1994; Eley et al., 2020), as well as to general psychopathology in the general population (Limburg et al., 2017). We must also keep in mind that these personality traits may interact with external factors, such as sociodemographic factors. Our results show that, not being in a marital or akin to marriage relationship was more frequent in those with depression and/or anxiety. This finding is in line with previous research linking single marital status as a risk factor for negative health outcomes, including physician’s suicide (Buckman et al., 2021; Rátiva Hernández et al., 2023). In the context of a culture of perfectionism and highly stressful endeavors, this finding seems to highlight the importance of meaningful human relationships for mental well-being.

In line with this reasoning, both perfectionism, work-related distress, and aggression from colleagues were significant for the final regression model. And, although the odds ratios were relatively small, the percentage of explained variance of each variable is considerable, given the complexity of the measured outcome. Thus, the interaction between these variables seems to us of particular importance: we believe it might reflect a learning environment where psychological safety is not present: “a group phenomenon defined as a shared belief held by teammates that it is safe to take interpersonal risks,” which, contrary to an environment of teaching by shaming, provides an environment where one’s mistakes can be acknowledged and views can be voiced without fearing embarrassment, judgment or repercussion. Environments where psychological safety is present have shown to improve the quality of learning, group connection and patient safety in healthcare facilities (Kumar, 2024). The fact that pediatricians are responsible for delivering care to children, gives rise to highly complex interactions as it awakens an innate need to protect and a heightened sense of responsibility in all people involved in the care of the sick child (both health-practitioners and parents), with implications both for the individual as well as the network (Goubert et al., 2008; Goubert and Simons, 2013). A high concern for errors might, in this sense, go hand in hand with the too normalized aggressiveness lived within this same culture, where an oppressive interpersonal climate will induce fear of failure (Fnais et al., 2014; Roberts, 2023; Kumar, 2024). Indeed, both having a high concern for errors and having been aggressed by colleagues were associated with depression and anxiety. This could be yet another example of teaching by shaming, with a clear negative consequence of this approach to learning, and a reflection of the criticism of oneself and others inherent to perfectionism (Martin et al., 2022). In this regard, it is relevant to notice that although the most common assailants of aggressions were patient’s relatives and patients, the aggression coming from peers was the one associated to a negative mental health outcome in this sample, highlighting the possible role of shame and perfectionism in this process. Additionally, and beyond the impact of this teaching environment on residents’ mental health and its implication for the quality of care they will be able to provide, the lack of psychological safety will work against the desired outcome (i.e., improving children’s medical attention): interpersonal climates which inhibit speaking up with questions, concerns, and challenges, are less able to detect and learn from mistakes (Edmondson, 2004). Indeed, an environment which facilitates the recognition of mistakes while retaining high standards and accountability, improve learning (Kumar, 2024). Nevertheless, it is relevant to keep in mind that harsh self-criticism, worry of one’s own performance and feelings of worthlessness are common symptoms of depression and anxiety, and the present cross-sectional design cannot dismiss that these symptoms might be responsible for a greater score in perfectionism in the group of physicians with depression and/or anxiety.

Finally, being the resident in charge of a patient who died was, as expected, associated with depression and anxiety. A patient’s death is a major adverse event, even more so when that outcome involves vulnerable populations, such as children. As a species, we are naturally prone to protect children. When one is involved in the care of a child who dies, questioning one’s responsibility in the outcome is desirable. Coping mechanisms to prevent shame and judgment from overcoming the more useful objective observation, and compassionate learning are vital tools we can teach health personnel to deal with adverse events (Neff et al., 2020). Yet, this is rarely taught in medical residencies (Rhodes-Kropf et al., 2005).

There are limitations that need to be mentioned and considered before generalizing our results. First, the non-probabilistic sample approach of the study limits the generalization of our findings as it is an important bias that should be considered. It is possible that some residents lacked the motivation to participate in the survey and pediatric residents with a specific interest in mental health themes were more likely to answer the survey. Also, this study focused on depressive and anxiety symptoms as rated by participants and can hence differ from objective evaluations. In terms of self-identifying symptoms, it has been reported that physicians tend to under-or over-react to their symptoms due to the absence of proper objective clinical assessment (Jonsson et al., 2023). We cannot rule out that pediatricians with more severe depressive or anxious symptoms may be more likely to recall and/or report instances of having been the victim of aggression or the physician in charge of a patient who died, both of which are unrelated to the etiology of depression and/or anxiety. Considering this and the cross-sectional design of the study, it is important to highlight that the results of the study do not imply causality and only associations can be evidenced, with further studies needed to support these associations and longitudinal studies required to identify risk factors for depression and/or anxiety.

Additionally, other factors contributing to depression and anxiety related to specific scenarios which differ from one institution to another, even more so, from one country to another, need to be further studied. Also, we did not find a difference in the total worked hours per day between groups. This variable was not evaluated in terms of the common work schedule, but this should not imply that unrestricted working schedules have no association with these outcomes; a possible explanation is that all residents in our sample could be exposed to long working hours.

In conclusion, pediatricians in training represent a population at risk for mental health problems. As the most important factor, residents with a history of previous depressive and anxious episodes should be closely monitored from their entry into residency to promote early intervention. Also, the culture promoting perfectionism, especially when related to internal and external judging and shaming, leading to aggressiveness toward oneself and colleagues, should be revisited. A more compassionate approach in a field with so many adversities will certainly entail better outcomes for both physicians and society as a whole.

## Data availability statement

The raw data supporting the conclusions of this article will be made available by the authors, without undue reservation.

## Ethics statement

The studies involving humans were approved by Ethics Committee of the Ramón de la Fuente Muñiz National Institute of Psychiatry. The studies were conducted in accordance with the local legislation and institutional requirements. The ethics committee/institutional review board waived the requirement of written informed consent for participation from the participants or the participants’ legal guardians/next of kin because informed consent was obtained by including the necessary information at the beginning of the online survey applied to the participants.

## Author contributions

MY-N: Validation, Methodology, Writing – review & editing, Writing – original draft, Conceptualization. DG-S: Resources, Data curation, Writing – review & editing, Writing – original draft, Validation, Methodology. RR-G: Investigation, Formal analysis, Writing – review & editing, Writing – original draft, Methodology. C-AT-Z: Data curation, Writing – review & editing, Writing – original draft, Methodology, Investigation, Formal analysis. R-AS-Á: Supervision, Resources, Project administration, Conceptualization, Writing – review & editing, Methodology. IN-C: Validation, Data curation, Writing – review & editing, Methodology, Conceptualization. A-CH-d-T: Supervision, Resources, Investigation, Writing – review & editing, Validation, Methodology, Data curation. AF: Writing – original draft, Project administration, Formal analysis, Conceptualization, Writing – review & editing.
